# 2-Chloro­methyl-2,3-dihydro­thieno[3,4-*b*][1,4]dioxine

**DOI:** 10.1107/S1600536809007156

**Published:** 2009-03-06

**Authors:** Jian Xu, Hao Xu, Ji-cai Quan, Fei Sha, Cheng Yao

**Affiliations:** a College of Science, Nanjing University of Technology, Xinmofan Road No. 5, Nanjing 210009, People’s Republic of China

## Abstract

In the mol­ecule of the title compound, C_7_H_7_ClO_2_S, the six-membered ring adopts a twisted conformation. In the crystal structure, weak inter­molecular C—H⋯O hydrogen bonds link the mol­ecules. There is also a weak C—H⋯π inter­action.

## Related literature

For a related structure, see: Jose *et al.* (2005 ▶). For bond-length data, see: Allen *et al.* (1987 ▶). For ring-puckering parameters, see: Cremer & Pople (1975 ▶).
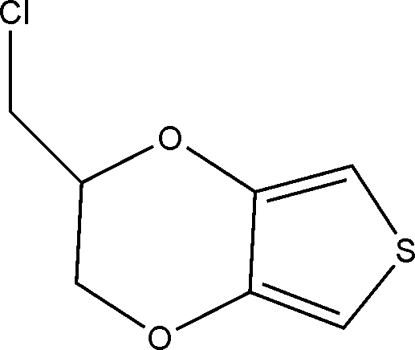


## Experimental

### 

#### Crystal data

C_7_H_7_ClO_2_S
*M* *_r_* = 190.64
Monoclinic, 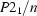

*a* = 10.227 (2) Å
*b* = 5.7500 (12) Å
*c* = 14.376 (3) Å
β = 105.55 (3)°
*V* = 814.4 (3) Å^3^
*Z* = 4
Mo *K*α radiation
μ = 0.67 mm^−1^
*T* = 294 K
0.30 × 0.20 × 0.10 mm

#### Data collection

Enraf–Nonius CAD-4 diffractometer
Absorption correction: ψ scan (North *et al.*, 1968 ▶) *T*_min_ = 0.825, *T*_max_ = 0.936
1565 measured reflections
1479 independent reflections
1065 reflections with *I* > 2σ(*I*)
*R*_int_ = 0.067
3 standard reflections frequency: 120 min intensity decay: 1%

#### Refinement

*R*[*F* ^2^ > 2σ(*F* ^2^)] = 0.065
*wR*(*F* ^2^) = 0.185
*S* = 1.01
1479 reflections
100 parameters
H-atom parameters constrained
Δρ_max_ = 0.41 e Å^−3^
Δρ_min_ = −0.29 e Å^−3^

### 

Data collection: *CAD-4 Software* (Enraf–Nonius, 1989 ▶); cell refinement: *CAD-4 Software*; data reduction: *XCAD4* (Harms & Wocadlo, 1995 ▶); program(s) used to solve structure: *SHELXS97* (Sheldrick, 2008 ▶); program(s) used to refine structure: *SHELXL97* (Sheldrick, 2008 ▶); molecular graphics: *ORTEP-3 for Windows* (Farrugia, 1997 ▶) and *PLATON* (Spek, 2009 ▶); software used to prepare material for publication: *SHELXTL* (Sheldrick, 2008 ▶).

## Supplementary Material

Crystal structure: contains datablocks I, global. DOI: 10.1107/S1600536809007156/hk2630sup1.cif

Structure factors: contains datablocks I. DOI: 10.1107/S1600536809007156/hk2630Isup2.hkl

Additional supplementary materials:  crystallographic information; 3D view; checkCIF report

## Figures and Tables

**Table 1:** Hydrogen-bond geometry (Å, °)

*D*—H⋯*A*	*D*—H	H⋯*A*	*D*⋯*A*	*D*—H⋯*A*
C2—H2*A*⋯O1^i^	0.98	2.45	3.317 (5)	146
C1—H1*B*⋯*Cg*1^ii^	0.97	2.75	3.708 (5)	168

Symmetry codes: (i) 
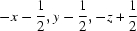
; (ii) 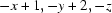. *Cg*1 is the centroid of the S/C4–C7 ring.

**Fig. 1.**
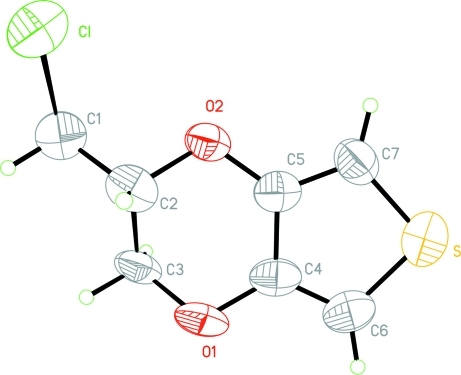
The molecular structure of the title molecule, with the atom-numbering scheme. Displacement ellipsoids are drawn at the 50% probability level.

**Fig. 2.**
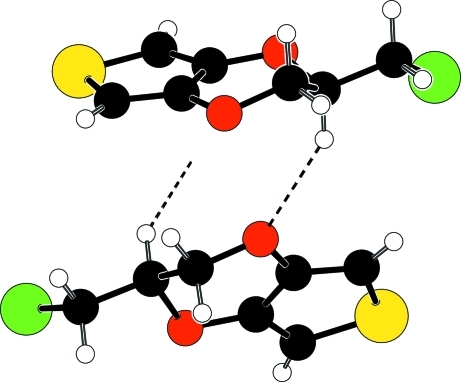
A partial packing diagram of the title compound. Hydrogen bonds are shown as dashed lines.

## supplementary crystallographic information

### Comment

A great deal of interest has been devoted in recent years to the synthesis and investigation of functionalized 3,4-ethylenedioxythiophene (EDOT) systems because of their potential as active materials in applications such as light-emitting diodes (OLEDs), plastic lasers, field-effect transistors and photovoltaic devices (Jose *et al.*, 2005). The title compound is an important intermediate in the synthesis of functionalized 3,4-ethylenedioxy- thiophene (EDOT) systems, and we report herein its crystal structure.

In the molecule of the title compound (Fig. 1), the bond lengths (Allen *et al.*, 1987) and angles are within normal ranges. Ring B (S/C4-C7) is, of course, planar. Ring A (O1/O2/C2-C5) is not planar, having total puckering amplitude, Q_T_, of 0.659 (3) Å and twisted conformation [φ = -149.80 (3)° and θ = 154.28 (3)°] (Cremer & Pople, 1975).

In the crystal structure, intermolecular C-H···O hydrogen bonds (Table 1) link the molecules (Fig. 2), in which they may be effective in the stabilization of the structure. There is also a weak C—H···π interaction (Table 1).

### Experimental

For the preparation of the title compound, 3,4-dimethoxythiophene (1.14 g, 7.9 mmol), 3-chloro-1,2-propanediol (2.45 g, 22.2 mmol), *p*-toluene- sulfonic acid monohydrate (0.151 g, 0.81 mmol) and dry toluene (27 ml) were added into a three necked flask equipped with an argon purge. The mixture was refluxed for 12 h. Then, diol (2.45 g, 22.2 mmol) was added and refluxed for 3 h. It was allowed to cool to room temperature. After removal of the solvent, the remaining crude product was isolated by flash chromatography [silica gel, hexane/dichloromethane (8:2)] to isolate the title compound, as a colorless solid (yield; 58%) (Jose *et al.*, 2005). Crystals suitable for X-ray analysis were obtained by dissolving the title compound (0.1 g) in hexane (25 ml) and evaporating the solvent slowly at room temperature for about 3 d.

### Refinement

H atoms were positioned geometrically, with C-H = 0.93, 0.98 and 0.97 Å for aromatic, methine and methylene H, respectively, and constrained to ride on their parent atoms, with U_iso_(H) = 1.2U_eq_(C).

### Crystal data

**Table:**

C_7_H_7_ClO_2_S	*F*(000) = 392
*M**_r_* = 190.64	*D*_x_ = 1.555 Mg m^−^^3^
Monoclinic, *P*2_1_/*n*	Mo *K*α radiation, λ = 0.71073 Å
Hall symbol: -P 2yn	Cell parameters from 25 reflections
*a* = 10.227 (2) Å	θ = 10–13°
*b* = 5.7500 (12) Å	µ = 0.67 mm^−^^1^
*c* = 14.376 (3) Å	*T* = 294 K
β = 105.55 (3)°	Block, colorless
*V* = 814.4 (3) Å^3^	0.30 × 0.20 × 0.10 mm
*Z* = 4	

### Data collection

**Table:**

Enraf–Nonius CAD-4 diffractometer	1065 reflections with *I* > 2σ(*I*)
Radiation source: fine-focus sealed tube	*R*_int_ = 0.067
graphite	θ_max_ = 25.4°, θ_min_ = 2.2°
ω/2θ scans	*h* = 0→12
Absorption correction: ψ scan (North *et al.*, 1968)	*k* = 0→6
*T*_min_ = 0.825, *T*_max_ = 0.936	*l* = −17→16
1565 measured reflections	3 standard reflections every 120 min
1479 independent reflections	intensity decay: 1%

### Refinement

**Table:**

Refinement on *F*^2^	Primary atom site location: structure-invariant direct methods
Least-squares matrix: full	Secondary atom site location: difference Fourier map
*R*[*F*^2^ > 2σ(*F*^2^)] = 0.065	Hydrogen site location: inferred from neighbouring sites
*wR*(*F*^2^) = 0.185	H-atom parameters constrained
*S* = 1.01	*w* = 1/[σ^2^(*F*_o_^2^) + (0.1*P*)^2^ + 0.92*P*] where *P* = (*F*_o_^2^ + 2*F*_c_^2^)/3
1479 reflections	(Δ/σ)_max_ < 0.001
100 parameters	Δρ_max_ = 0.41 e Å^−^^3^
0 restraints	Δρ_min_ = −0.29 e Å^−^^3^

### Special details

**Table:**

Geometry. All e.s.d.'s (except the e.s.d. in the dihedral angle between two l.s. planes) are estimated using the full covariance matrix. The cell e.s.d.'s are taken into account individually in the estimation of e.s.d.'s in distances, angles and torsion angles; correlations between e.s.d.'s in cell parameters are only used when they are defined by crystal symmetry. An approximate (isotropic) treatment of cell e.s.d.'s is used for estimating e.s.d.'s involving l.s. planes.
Refinement. Refinement of *F*^2^ against ALL reflections. The weighted *R*-factor *wR* and goodness of fit *S* are based on *F*^2^, conventional *R*-factors *R* are based on *F*, with *F* set to zero for negative *F*^2^. The threshold expression of *F*^2^ > σ(*F*^2^) is used only for calculating *R*-factors(gt) *etc*. and is not relevant to the choice of reflections for refinement. *R*-factors based on *F*^2^ are statistically about twice as large as those based on *F*, and *R*- factors based on ALL data will be even larger.

### Fractional atomic coordinates and isotropic or equivalent isotropic displacement parameters (Å^2^)

**Table:**

	*x*	*y*	*z*	*U*_iso_*/*U*_eq_	
Cl	−0.37084 (12)	0.1937 (2)	0.51615 (9)	0.0759 (5)	
S	0.12047 (12)	0.1840 (2)	0.27636 (9)	0.0740 (5)	
O1	−0.1425 (3)	0.6480 (5)	0.29741 (19)	0.0619 (8)	
O2	−0.1277 (2)	0.2762 (4)	0.43358 (19)	0.0505 (7)	
C1	−0.2959 (4)	0.4560 (8)	0.4923 (3)	0.0569 (10)	
H1A	−0.3636	0.5786	0.4805	0.068*	
H1B	−0.2238	0.4991	0.5486	0.068*	
C2	−0.2397 (4)	0.4363 (7)	0.4079 (3)	0.0524 (9)	
H2A	−0.3096	0.3729	0.3531	0.063*	
C3	−0.1903 (4)	0.6650 (6)	0.3787 (3)	0.0539 (10)	
H3A	−0.2641	0.7765	0.3663	0.065*	
H3B	−0.1181	0.7236	0.4320	0.065*	
C4	−0.0539 (4)	0.4634 (7)	0.3034 (3)	0.0515 (9)	
C5	−0.0507 (4)	0.2828 (6)	0.3683 (3)	0.0477 (9)	
C6	0.0309 (4)	0.4363 (8)	0.2481 (3)	0.0632 (11)	
H6A	0.0400	0.5405	0.2008	0.076*	
C7	0.0424 (4)	0.1179 (7)	0.3626 (3)	0.0591 (10)	
H7A	0.0605	−0.0143	0.4012	0.071*	

### Atomic displacement parameters (Å^2^)

**Table:**

	*U*^11^	*U*^22^	*U*^33^	*U*^12^	*U*^13^	*U*^23^
Cl	0.0794 (8)	0.0787 (9)	0.0741 (8)	−0.0128 (6)	0.0285 (6)	−0.0007 (6)
S	0.0758 (8)	0.0812 (9)	0.0696 (8)	0.0012 (6)	0.0275 (6)	−0.0095 (6)
O1	0.0693 (18)	0.0601 (18)	0.0461 (16)	0.0112 (14)	−0.0024 (13)	0.0218 (13)
O2	0.0571 (15)	0.0440 (14)	0.0484 (15)	0.0044 (12)	0.0110 (12)	0.0090 (12)
C1	0.061 (2)	0.066 (3)	0.0370 (19)	−0.005 (2)	0.0014 (17)	−0.0062 (18)
C2	0.051 (2)	0.053 (2)	0.042 (2)	−0.0049 (17)	−0.0081 (16)	−0.0067 (17)
C3	0.068 (2)	0.047 (2)	0.040 (2)	0.0146 (18)	0.0034 (17)	0.0132 (17)
C4	0.055 (2)	0.057 (2)	0.0329 (18)	−0.0073 (18)	−0.0059 (16)	0.0042 (17)
C5	0.059 (2)	0.044 (2)	0.0355 (18)	−0.0114 (17)	0.0042 (15)	−0.0069 (16)
C6	0.069 (3)	0.073 (3)	0.044 (2)	−0.004 (2)	0.0078 (19)	0.006 (2)
C7	0.064 (2)	0.051 (2)	0.059 (2)	0.0111 (19)	0.012 (2)	0.001 (2)

### Geometric parameters (Å, °)

**Table:**

Cl—C1	1.766 (4)	C2—C3	1.508 (5)
S—C7	1.688 (4)	C2—H2A	0.9800
S—C6	1.706 (5)	C3—H3A	0.9700
O1—C4	1.384 (5)	C3—H3B	0.9700
O1—C3	1.386 (5)	C4—C6	1.333 (5)
O2—C5	1.377 (5)	C4—C5	1.391 (5)
O2—C2	1.439 (5)	C5—C7	1.362 (5)
C1—C2	1.480 (5)	C6—H6A	0.9300
C1—H1A	0.9700	C7—H7A	0.9300
C1—H1B	0.9700		
			
C7—S—C6	92.1 (2)	C2—C3—H3A	108.9
C4—O1—C3	112.0 (3)	O1—C3—H3B	108.9
C5—O2—C2	111.6 (3)	C2—C3—H3B	108.9
C2—C1—Cl	112.2 (3)	H3A—C3—H3B	107.8
C2—C1—H1A	109.2	C6—C4—O1	124.9 (4)
Cl—C1—H1A	109.2	C6—C4—C5	114.3 (4)
C2—C1—H1B	109.2	O1—C4—C5	120.8 (3)
Cl—C1—H1B	109.2	C7—C5—O2	124.0 (3)
H1A—C1—H1B	107.9	C7—C5—C4	111.7 (4)
O2—C2—C1	107.2 (3)	O2—C5—C4	124.3 (3)
O2—C2—C3	109.0 (3)	C4—C6—S	110.5 (3)
C1—C2—C3	113.2 (3)	C4—C6—H6A	124.7
O2—C2—H2A	109.1	S—C6—H6A	124.7
C1—C2—H2A	109.1	C5—C7—S	111.3 (3)
C3—C2—H2A	109.1	C5—C7—H7A	124.3
O1—C3—C2	113.2 (3)	S—C7—H7A	124.3
O1—C3—H3A	108.9		
			
C5—O2—C2—C1	−167.2 (3)	C6—C4—C5—C7	1.5 (5)
C5—O2—C2—C3	−44.3 (4)	O1—C4—C5—C7	179.5 (3)
Cl—C1—C2—O2	−66.6 (3)	C6—C4—C5—O2	178.7 (3)
Cl—C1—C2—C3	173.1 (3)	O1—C4—C5—O2	−3.2 (5)
C4—O1—C3—C2	−48.0 (4)	O1—C4—C6—S	−178.8 (3)
O2—C2—C3—O1	63.1 (4)	C5—C4—C6—S	−0.8 (5)
C1—C2—C3—O1	−177.6 (3)	C7—S—C6—C4	0.0 (3)
C3—O1—C4—C6	−163.6 (4)	O2—C5—C7—S	−178.7 (3)
C3—O1—C4—C5	18.6 (5)	C4—C5—C7—S	−1.4 (4)
C2—O2—C5—C7	−165.2 (4)	C6—S—C7—C5	0.8 (3)
C2—O2—C5—C4	17.9 (5)		

### Hydrogen-bond geometry (Å, °)

**Table:**

*D*—H···*A*	*D*—H	H···*A*	*D*···*A*	*D*—H···*A*
C2—H2A···O1^i^	0.98	2.45	3.317 (5)	146
C1—H1B···Cg1^ii^	0.97	2.75	3.708 (5)	169

Symmetry codes: (i) −*x*−1/2, *y*−1/2, −*z*+1/2; (ii) −*x*+1, −*y*+2, −*z*.
